# A Rare Convergence: Verrucous Squamous Cell Carcinoma Arising From Bowen’s Disease in an Uncommon Cutaneous Location

**DOI:** 10.7759/cureus.99899

**Published:** 2025-12-22

**Authors:** Anupriyaa A, Sreenidhi Sreeram, Leena Joseph, Balasubramanian Venkitaraman

**Affiliations:** 1 College of Medicine, Sri Ramachandra Institute of Higher Education and Research, Chennai, IND; 2 Pathology, Sri Ramachandra Institute of Higher Education and Research, Chennai, IND; 3 Surgical Oncology, Sri Ramachandra Institute of Higher Education and Research, Chennai, IND

**Keywords:** bowen’s disease, cutaneous squamous cell carcinoma, histopathology, rare skin tumor, skin neoplasms, squamous cell carcinoma, surgical excision, surgical flaps, verrucous carcinoma, wide local excision

## Abstract

Verrucous squamous cell carcinoma (VSCC) and Bowen’s disease (BD) are neoplasms of squamous cell origin. Bowen’s disease is the widely used term for squamous cell carcinoma in situ and primarily involves the skin, whereas VSCC represents a well-differentiated, locally aggressive variant of squamous cell carcinoma. These entities differ in their risk factors, biological behavior, and therapeutic approach. Cutaneous VSCC is typically human papillomavirus-independent (HPV-independent) and often requires wide local excision, while BD is frequently associated with HPV infection, arises on sun-exposed skin, and may respond to non-surgical treatment modalities. We report a rare case of VSCC arising in a background of Bowen’s disease over the lower back--an uncommon site for both lesions. The patient underwent wide local excision with Limberg flap reconstruction, and histopathological examination confirmed VSCC with adjacent Bowen’s disease. This case highlights the importance of recognizing dual pathology in atypical cutaneous lesions to ensure appropriate surgical planning and management.

## Introduction

Verrucous variant of squamous cell carcinoma (VSCC) is an uncommon, well-differentiated, indolent yet locally aggressive subtype of squamous cell carcinoma that can arise from both cutaneous and mucosal surfaces. In contrast, Bowen’s disease (BD) represents squamous cell carcinoma in situ and primarily affects the skin.

VSCC most commonly involves mucosal sites, such as the buccal mucosa, tongue, and glottic region of the larynx, predominantly affecting the head and neck region, whereas cutaneous involvement is relatively rare. BD, on the other hand, frequently affects the head and neck as well as the extremities. A rare genital variant, erythroplasia of Queyrat, presents as carcinoma in situ of the penile mucosa [1]. BD predominantly involves the cheeks and lower limbs in women, while in men, the bald scalp and ear are more commonly affected [1].

BD is associated with a broad spectrum of risk factors, including chronic ultraviolet exposure, ionizing radiation, carcinogens such as arsenic, viral infections--most notably high-risk human papillomavirus (HPV) subtypes 16 and 18 and Merkel cell polyomavirus--autoimmune disorders, such as Sjögren’s syndrome, immunosuppression (particularly in organ transplant recipients, with risk varying by transplant type: heart > kidney > liver), chronic trauma, certain medications (corticosteroids, azathioprine, and cyclosporine), and genetic predisposition [2,3].

Risk factors for cutaneous VSCC include chronic inflammation and irritation, particularly in long-standing scars or non-healing wounds. In contrast, mucosal VSCC has been associated with low-risk HPV subtypes (HPV 6 and 11), tobacco use, alcohol consumption, and other local irritants.

Herein, we report a unique case of VSCC arising in a background of BD on the right lower back--an uncommon site for both entities. This case highlights the clinical, histopathological, and surgical aspects of this rare convergence, which, to our knowledge, has not been previously reported in the literature.

## Case presentation

An 85-year-old female presented with a hyperpigmented growth surrounded by a depigmented area of skin with scaling and excoriation over the right lower back. The lesion had been progressively enlarging for one and a half years, with occasional mild bleeding. Her medical history included systemic hypertension and a cerebrovascular accident two years prior. She reported no pain, fever, malaise, weight loss, trauma, prior raw areas, long-term drug use, including immunosuppressive medications, arsenic exposure, or similar lesions elsewhere on the body.

**Figure 1:**
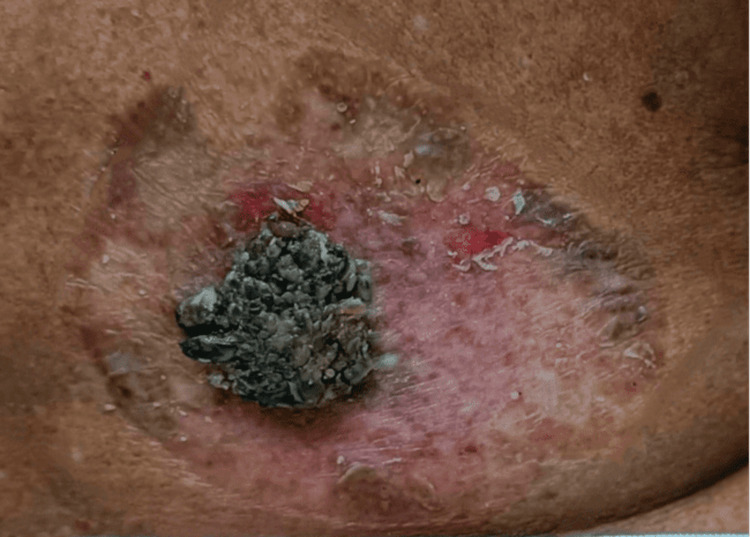
Clinical image Clinical image showing a 3 cm × 2 cm hyperpigmented growth, surrounded by a 5 cm × 8 cm area of depigmented skin with scaling and excoriation

On clinical examination, a 3 cm × 2 cm hyperpigmented growth was observed, surrounded by a 5 cm × 8 cm area of depigmented, scaly, and excoriated skin at the level of the iliac crest (Figure 1). No regional lymphadenopathy was noted.

Routine preoperative investigations, including complete blood count, renal and liver function tests, and coagulation profile, were within normal limits. Imaging was not performed due to the superficial nature of the lesion and absence of clinical lymphadenopathy. Given the high suspicion of skin malignancy, the patient underwent a wide local excision with a two-centimeter margin clearance, including the fascia overlying the muscle. Superior, medial, and inferolateral margins were included to ensure complete clearance. Reconstruction was achieved using a Limberg flap, providing satisfactory cosmetic and functional outcomes.

**Figure 2:**
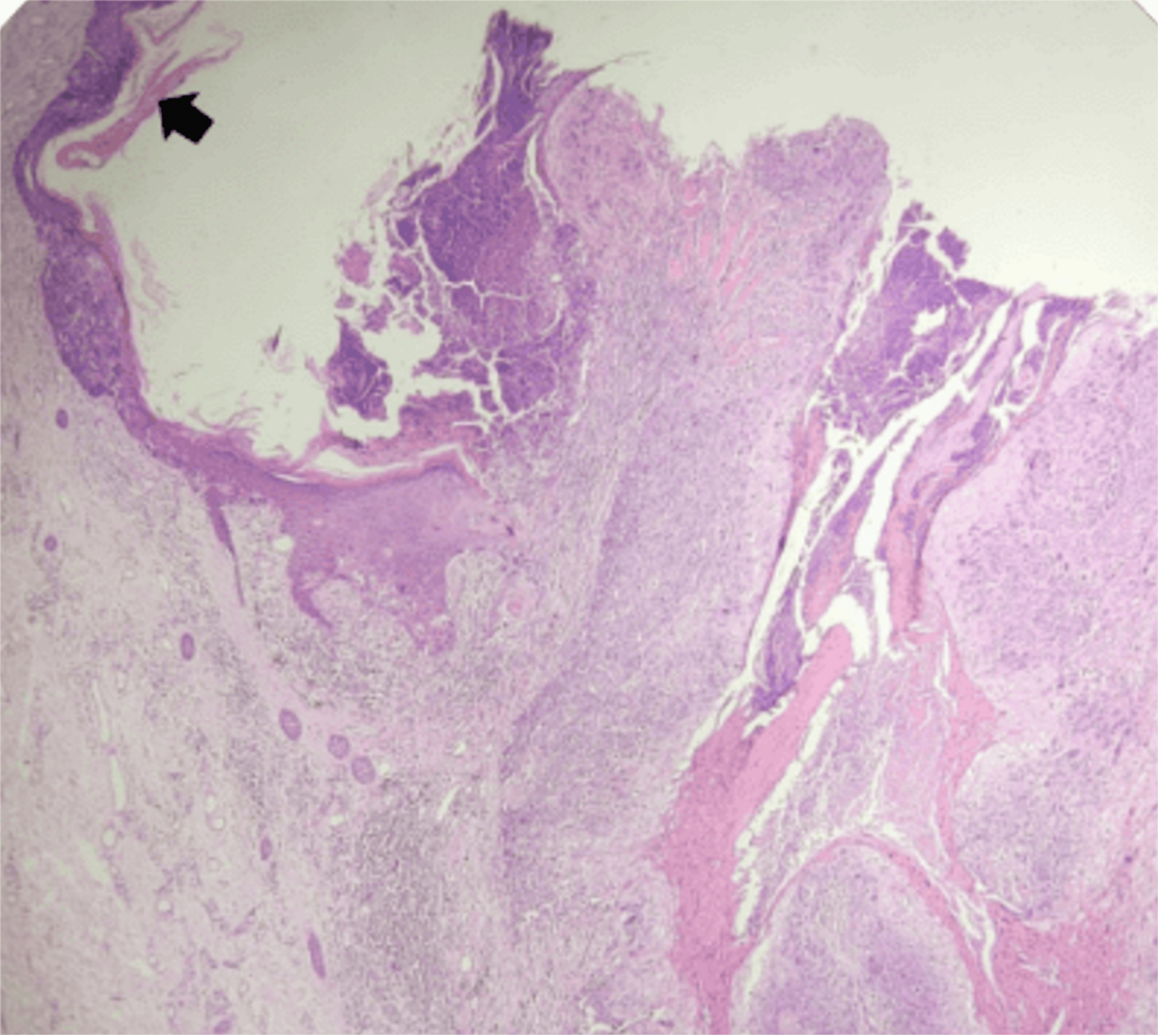
H&E (x200) with annotation Histopathology image annotated with an arrow highlighting the region of Bowen’s disease, with evident acanthosis (Hematoxylin and Eosin (H&E) stain, ×200).

**Figure 3:**
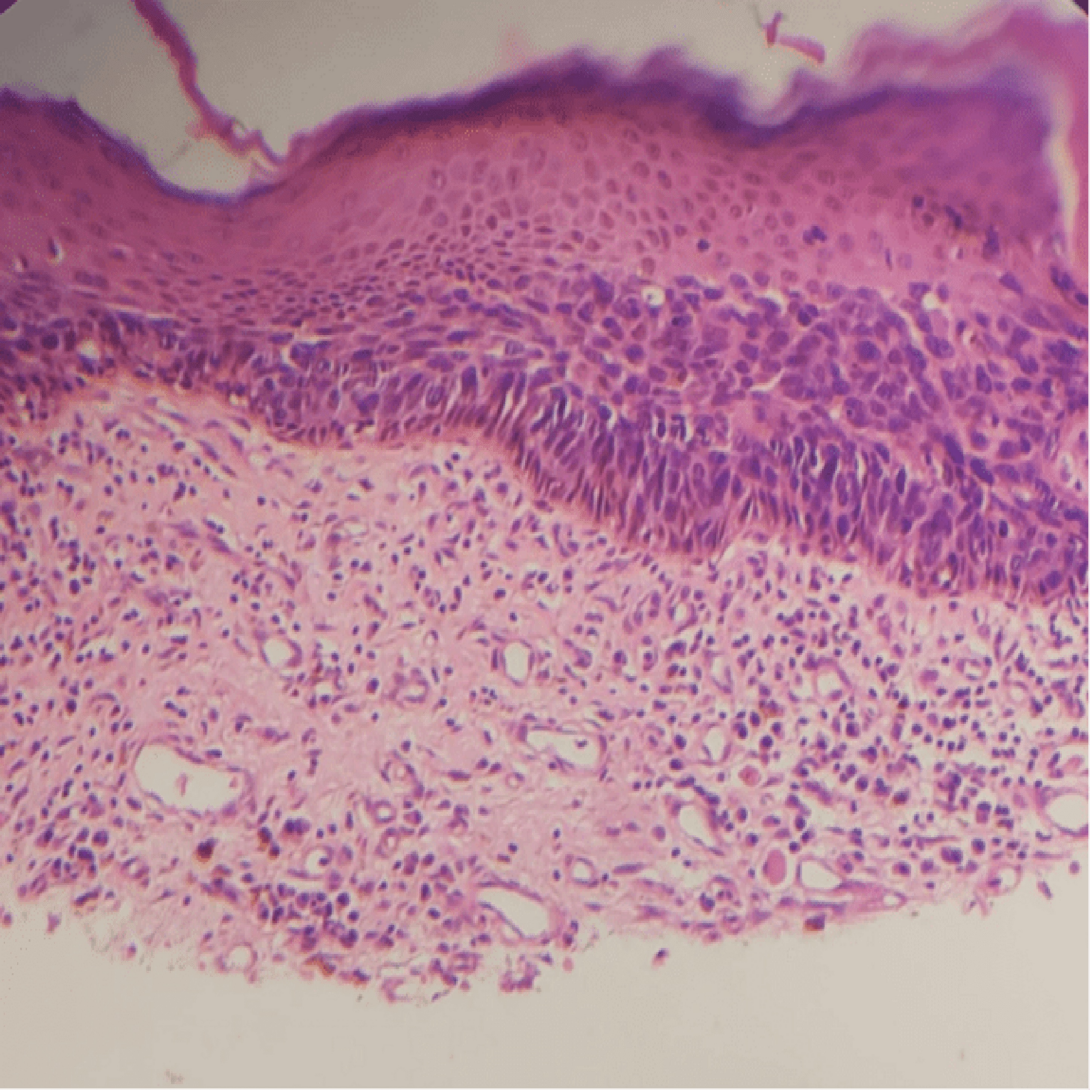
H&E (x400) Histopathology image of the underlying dermis showing lymphocytic infiltration (Hematoxylin and Eosin (H&E) stain, ×400).

Gross examination revealed a lesion with a thickness of 6 mm and a depth of invasion of 3 mm. Histopathological analysis confirmed verrucous squamous cell carcinoma with adjacent full-thickness atypia of the squamous epithelium, consistent with Bowen’s disease (Figures 2, 3). No conventional invasive squamous cell carcinoma component, lymphovascular invasion, or perineural invasion was observed. All surgical margins were free of tumor, corresponding to Stage I disease.

The postoperative course was uneventful, with wound healing progressing without infection, dehiscence, or flap necrosis. Follow-up evaluations at 1, 3, 6, and 12 months revealed no recurrence, regional lymphadenopathy, or new lesions. The Limberg flap yielded favorable cosmetic and functional outcomes. At one year, the patient remained disease-free and was advised to maintain regular follow-up, sun protection, self-examinations, and to promptly report any new lesions or changes in the skin.

## Discussion

VSCC is an uncommon, well-differentiated variant of squamous cell carcinoma characterized by slow growth, an exophytic wart-like appearance, and pronounced local invasion with a low propensity for metastasis [1-5]. Clinically, VSCC typically presents as a pebbly or micronodular lesion, often resembling a benign verrucous growth, which may delay diagnosis [1,2,4]. Despite its indolent behavior, the tumor can be locally destructive if not adequately treated.

Histopathologically, VSCC is distinguished by thick parakeratinized epithelium, broad and bulbous rete ridges, minimal cytological atypia, and a pushing rather than infiltrative growth pattern, with preservation of the basement membrane. A prominent chronic inflammatory infiltrate, predominantly lymphocytic, is frequently observed in the underlying stroma [2,3,5]. These features differentiate VSCC from conventional invasive squamous cell carcinoma, which typically demonstrates greater cytological atypia, infiltrative growth, and a higher metastatic potential [6].

BD, the cutaneous form of squamous cell carcinoma in situ, presents clinically as a well-demarcated erythematous, scaly patch or plaque that may be asymptomatic or pruritic [7,8]. Histologically, BD is characterized by full-thickness epidermal atypia with a classic “windblown” appearance, acanthosis, elongated rete ridges, dyskeratotic keratinocytes, and an intact basement membrane, often accompanied by lymphocytic infiltration in the dermis [7]. These features reflect its intraepidermal malignant potential without dermal invasion.

Although both VSCC and BD originate from squamous epithelium, their etiological factors and biological behavior differ. Cutaneous VSCC is typically HPV-independent and is more commonly associated with chronic inflammation, irritation, or long-standing lesions, whereas BD has been linked to ultraviolet radiation exposure, immunosuppression, and viral oncogenesis, particularly high-risk HPV subtypes [1,8,9]. VSCC more frequently affects elderly males, while BD shows a higher prevalence in elderly females [1,4].

Management strategies for these entities also differ significantly. VSCC generally requires wide local excision with adequate margins due to its locally aggressive nature, and reconstructive procedures may be necessary depending on tumor size and location [1,2,3]. Radiotherapy has a limited role and is reserved for select cases [2]. In contrast, BD can often be managed with non-surgical modalities, such as topical agents (imiquimod, 5-fluorouracil), photodynamic therapy, cryotherapy, curettage with cautery, laser therapy, or surgical excision, depending on lesion characteristics and patient factors [9,10].

The coexistence of VSCC arising in a background of BD is exceedingly rare. In our case, histopathology confirmed VSCC with adjacent full-thickness atypia consistent with BD, without evidence of conventional invasive squamous cell carcinoma, lymphovascular invasion, or perineural invasion. Differential diagnoses considered included conventional invasive SCC, keratoacanthoma, and verrucous hyperplasia. Accurate histopathological diagnosis was critical, as management strategies differ for VSCC and BD.

Although direct reports of VSCC arising in BD are limited, similar cases of verrucous carcinoma developing in pre-existing epidermal lesions or chronic inflammatory skin conditions have been reported [2,3,5], highlighting the need for vigilance in long-standing atypical cutaneous lesions. This case underscores the importance of maintaining a high index of suspicion for dual pathology, as recognition significantly impacts treatment planning, prognosis, and long-term surveillance.

## Conclusions

This case highlights a rare occurrence of verrucous squamous cell carcinoma arising from Bowen’s disease in an atypical cutaneous location. Early recognition, appropriate surgical intervention, and thorough histopathological evaluation are key to achieving favorable outcomes. Long-term follow-up is essential due to the risk of recurrence or the development of new lesions. Clinicians should maintain a high index of suspicion for dual pathology when evaluating atypical skin lesions.
